# Modulatory Effects of *Lactarius hatsudake* on Obesity and Gut Microbiota in High-Fat Diet-Fed C57BL/6 Mice

**DOI:** 10.3390/foods13060948

**Published:** 2024-03-20

**Authors:** Hanyu Zhu, Tao Hou

**Affiliations:** 1 College of Life Science, Hengyang Normal University, Hengyang 421000, China; zhuhzau@163.com; 2 College of Food Science and Technology, Huazhong Agricultural University, Wuhan 430070, China

**Keywords:** *Lactarius hatsudake*, biochemical index, gut microbiota, correlation analysis, obesity

## Abstract

*Lactarius hatsudake* (LH), a great wild endemic fungus, contains rich nutritional components with medicinal properties. The effects of LH on body weight, liver weight, liver injury, blood lipids, and gut microbiota in C57BL/6 mice fed a high-fat diet (HFD) for 8 weeks was examined in this research. Though there was no clear impact on weight loss, the findings indicate that LH treatment effectively decreased liver damage caused by HFD, as well as lowered serum total cholesterol, triacylglycerol, and low-density lipoprotein cholesterol levels. Additionally, it positively influenced gut microbiota to resemble that of mice on a normal diet. In HFD-fed mice, LH markedly boosted the levels of *Parabacteroides*, unclassified *Muribaculaceae*, *Oscillibacter*, and unclassified *Oscillospiraceae*, while reducing the abundance of *Lachnospiraceae NK4A136* group and *Erysipelatoclostridium*, as well as the ratio of Firmicutes to Bacteroidetes. Further analysis of correlation indicate a possible connection between obesity and gut microbiota. Obesity-related indices show a positive association with unclassified *Eubacterium coprostanoligenes* group, *Blautia*, and *Erysipelatoclostridium*, while displaying a negative correlation with unclassified *Muribaculaceae*, unclassified *Clostridia vadinBB60* group, *Helicobacter*, *Oscillibacter*, unclassified *Ruminococcaceae*, *Parabacteroides*, and unclassified *Oscillospiraceae*. The results suggest that LH can help combat obesity and may have the potential to be utilized as a functional food.

## 1. Introduction

Obesity is a glucose and lipid metabolic disease involving the abnormal buildup of fat in the body, leading to various health problems, including hypertension, hyperlipidemia, cardiovascular disease, and type 2 diabetes [1,2]. There are now almost 2 billion overweight adults in the world, and this trend continues to worsen, making obesity a global epidemic [3]. Obesity and its aggravation of a number of major diseases have become an issue of public health threat, which negatively impacts the quality and longevity of human life and the cost of healthcare [4,5]. Thus, it is a major challenge for us to prevent or ameliorate obesity.

The gut microbiota, often called the “forgotten organ” or “new organ” of hosts, has a strong connection to their overall well-being [6,7]. Multiple research studies have suggested that a variety of human illnesses, such as obesity and related metabolic disorders, are linked to imbalances in gut microbiota [8,9]. The diet plays a significant role in determining the makeup of gut bacteria [10]. The association among diet, gut microbiota, and host health has been elegantly demonstrated in animal models. For instance, switching animal diets from low-fat to high-fat leads to a decrease in Bacteroides and an increase in Firmicutes in the gut microbiota, which is associated with higher energy absorption, fat accumulation, and ultimately gut inflammation and permeability [11]. Research has demonstrated that certain food items and their active ingredients (like antibiotics, prebiotics, and probiotics) can be targeted to modify the makeup and arrangement of gut bacteria, potentially impeding the onset of obesity [2,4,5].

For centuries, mushrooms have been used for both medicinal and culinary purposes, providing a rich source of beneficial prebiotics with high levels of bioactive substances including polysaccharides, fibers, terpenes, polyphenols, sterols, flavonoids, and alkaloids [11,12]. The potential for edible mushrooms and their extracts to combat obesity has been studied both in laboratory settings and in living organisms. The aqueous extracts from *Ganoderma lucidum* mycelium [13] and *Antrodia cinnamomea* [14] were found to decrease body weight, fat storage, inflammation, insulin resistance, and the ratio of Firmicutes to Bacteroidetes in mice on a high-fat diet. Polysaccharides from *Pleurotus eryngii* [15] and *Dictyophora indusiate* [16] exhibited anti-obesity properties and altered the gut microbiota composition in obese mice. Additionally, chitosan from *Flammulina velutipes* helped prevent or treat dietary obesity by inhibiting fat digestion and absorption in the digestive system while also promoting fat breakdown in adipocytes [17]. Enhancing dietary patterns through the intake of mushrooms that have anti-obesity properties could be a successful strategy in combating obesity.

In our previous study, we found that the fruiting body of a wild *Lactarius hatsudake* (LH) contained high concentrations of total carbohydrate, protein, and essential elements, while being low in fat and calories [18], which revealed its potential use as a functional food ingredient. Although studies have been conducted on many mushrooms and their extracts that could have health impacts on the HFD-fed host, limited studies focus on how LH affects health or modulates the gut microbial composition. Consequently, this study aimed to examine how LH affects body and liver weight, blood lipid levels, liver histology, and gut microbiota in mice on a HFD, with the goal of determining its potential for reducing or treating obesity.

## 2. Materials and Methods

### 2.1. Materials

The fruiting body samples of LH were collected from the Nanyue mountainous region in China and freeze-dried. Dried LH was ground using a pulverizer and passed through a 120-mesh sieve to obtain powder. This strain has been identified in previous work, and the contents of total polysaccharides, protein, fat, and ash of LH were 66.43 ± 1.64, 24.10 ± 1.21, 2.50 ± 0.05, and 6.97 ± 0.16 g/100 g d.w. [18].

### 2.2. Animals and Experimental Design

**Figure 1:**
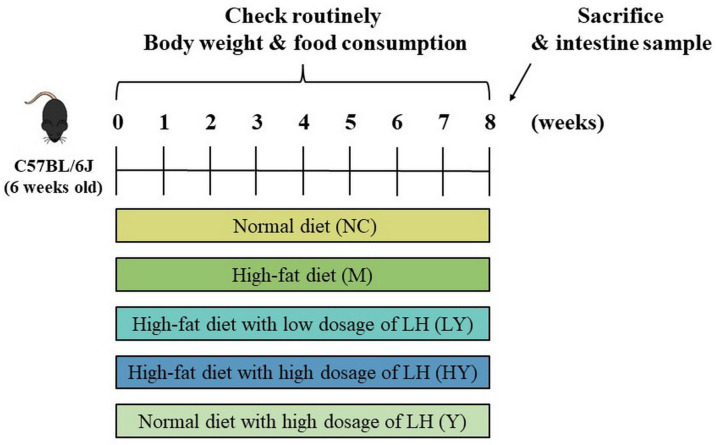
The experiment design of this study. Fifty mice were randomly divided into 5 groups with different kinds of diets: normal diet (NC), high-fat diet (M), high-fat diet with low dosage (2%, *w*/*w*) of LH (LY), high-fat diet with high dosage (5%, *w*/*w*) of LH (HY), normal diet with high dosage (5%, *w*/*w*) of LH (Y).

Fifty male C57BL/6J mice, aged 6 weeks, were obtained from the Experimental Animal Center of Huazhong Agricultural University with the laboratory animal permission number SYXK2020-0084. They were kept in a specific pathogen-free (SPF) animal facility with a 12 h light–dark cycle at a temperature of 24 ± 1 °C and a relative humidity of 30–40%. Water and food were free to access. Following a 3-day adjustment period, the mice were randomly divided into 5 groups (n = 10 per group), with varying diets: (1) normal diet (NC), (2) high-fat diet (M), (3) high-fat diet with low dosage (2%, *w*/*w*) of LH (LY), (4) high-fat diet with high dosage (5%, *w*/*w*) of LH (HY), and (5) normal diet with high dosage (5%, *w*/*w*) of LH (Y). The LH powder was added to each diet with the corresponding dosage. The normal diet (10% fat, 15% protein, 75% carbohydrate, 3.8 Kcal/g) and high-fat diet (60% fat, 15% protein, 25% carbohydrate, 5.5 Kcal/g) were purchased from Trophic Animal Feed High-tech Co., Ltd., Haian, China. A weekly record of food consumption and body weight was conducted. Following the 8-week experiment, all mice were deprived of food for 12 h before being euthanized by anesthesia with ether and cervical dislocation. Serum was obtained by collecting blood samples and centrifuging them at 3000× *g* for 15 min at 4 °C. The intestinal tract contents were obtained for intestinal microbiota analysis. The liver tissues were collected to weigh them. The schematics of the animal study design are shown in Figure 1. The experimental protocols and animal well-being, which included a reduction in the number of animals employed, were conducted in accordance with China’s Animal Care and Use Guidelines and authorized by the Animal Ethics Committee at Huazhong Agricultural University (Approval Number HZAUMO-2022-0123).

### 2.3. Biochemical Assays

Commercial enzymatic kits from Nanjing Jiancheng Bioengineering Institute Co. Ltd. (Nanjing, China) were used to analyze the levels of total cholesterol (TC), total triacylglycerol (TG), low-density lipoprotein cholesterol (LDL-C), and high-density lipoprotein cholesterol (HDL-C) in the blood serum following the manufacturer’s instructions. In brief, the enzyme was reacted with the sample, the standard, and distilled water (the blank), respectively. After incubation, the absorbance was determined, and the parameter was calculated as (absorbance of the sample − absorbance of the blank)/(absorbance of the standard − absorbance of the blank).

### 2.4. Histological Analysis

Liver samples were fixed in 4% paraformaldehyde, encased in paraffin, and sliced into sections approximately 4 μm thick. Following dewaxing, the sections were then stained using hematoxylin and eosin (H&E). Histological alterations in groups were examined using an optical microscope (Leica, Germany) and images were acquired.

### 2.5. DNA Extraction and Sequencing

Total genomic DNA from intestine samples (n = 5 per group) was extracted, and the V3–V4 hypervariable region of microbial 16S rRNA was amplified. The DNA quality and quantity were monitored on 1% agarose gels. Following PCR amplification using primers 341F and 806R, the Gel Extraction Kit (Qiagen, Hilden, Germany) was utilized for purification, and the resulting amplicons were sequenced on an Illumina Novaseq platform.

### 2.6. Bioinformatics Analysis 

After sequencing, the low-quality reads were filtered by Trimmomatic v0.33 and assembled to obtain clean reads. The DADA2 algorithm within the QIIME2 platform (Version QIIME2 2020.6) was employed for denoising the quality tags, followed by clustering the resulting initial ASVs into operational taxonomic units (OTUs). QIIME2 software was utilized for species annotation and taxonomic analysis, with the results visualized using R software (Version 3.1.1). The α-diversity was shown at the OTU level, whereas β-diversity was demonstrated through the Jaccard distance and visualized with PCoA. A relative abundance heatmap of the top 20 species at the genus level was generated in R software. LEfSe was used to determine variations between groups by combining the Kruskal–Wallis test to identify species with notable abundance differences across all groups and LDA to measure the impact of these distinct species. A Pearson correlation analysis was performed to examine the connection between gut microbiota and indices related to obesity. Finally, an analysis of the connection between the gut microbiome and factors related to obesity was conducted using redundancy analysis (RDA). 

### 2.7. Statistical Analysis

Results were shown as means ± standard deviation (SD). Data analysis was conducted using SPSS 23.0 software from SPSS Inc. in Chicago, IL, USA. Statistical significance for more than two groups was assessed using one-way analysis of variance (ANOVA) followed by the Duncan post hoc test. The results from the experiments that were conducted with two groups were analyzed with Student’s *t*-test. Statistically significant results were defined as *p*-values less than 0.05. The Pearson correlation coefficient *r* was used to examine the connections between obesity-related measures and the gut microbiota, with a significance level of *p* < 0.05. The *r* values can range from −1, indicating perfect anti-correlation, to 0, indicating no linear correlation, to +1, indicating perfect positive correlation.

## 3. Results and Discussion

### 3.1. Impact of LH on the Growth Condition and Weight of Mice

**Figure 2:**
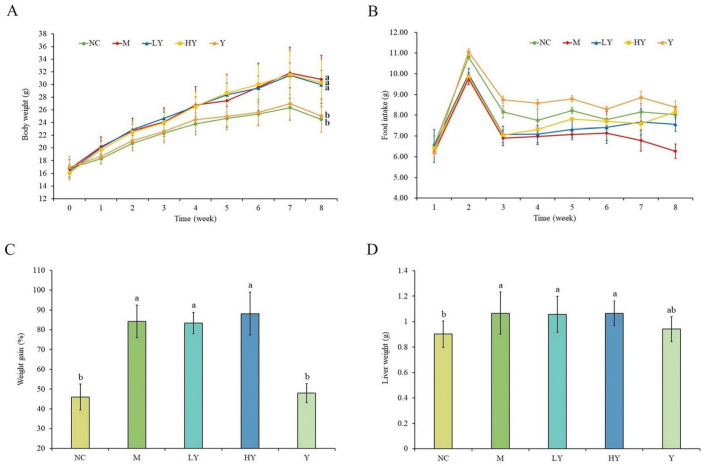
Effects of LH on body weight (**A**), food intake (**B**), weight gain (**C**), and liver weight (**D**) in mice. All data are shown as means ± SD (n = 10 per group). Different lowercase letters above the columns indicate statistically significant differences at *p* < 0.05 (one-way analysis of variance followed by Duncan post hoc test).

LH was administered as a dietary supplement to mice on a HFD in order to investigate its potential for inhibiting obesity in this study. As shown in Figure 2A, the body weight of C57BL/6 mice in different groups is presented. There was no observable difference for the initial weight among the groups, and the growth trend of body weight in the NC and Y groups was slower than that in the M group. By the conclusion of the 8th week, the M group exhibited significantly greater body weight and weight gain percentage compared to the NC group (*p* < 0.05), confirming the successful establishment of the obese mouse model. Nevertheless, adding LH to the high-fat diet did not result in any noticeable changes in body weight or weight gain, as shown by the LY and HY groups (Figure 2). Food consumption in the M groups was notably less than in the NC and Y groups starting from the second week (*p* < 0.05), yet both groups experienced weight gain in a comparable manner, indicating the effectiveness of a high-fat diet with increased caloric content (Figure 2B). Meanwhile, there was no observable change in the liver weight of mice in the M, LY, and HY groups (Figure 2D). These results implied that supplementation with 2% and 5% of LH did not have apparent influences on reducing body weight, weight gain, and liver weight in HFD-fed mice.

A growing body of research has demonstrated that edible mushrooms and their extracts can ameliorate obesity [19,20]. Consistent with findings in prior studies, adding whole *Agrocybe cylindracea* [5,19] and *Pleurotus ostreatus* [21] to the diet of mice fed a HFD resulted in decreased weight gain and liver weight in a manner that depended on the dosage. Conversely, a study found that consuming a combination of Japanese mushrooms at levels of 0.5% and 3% in a HFD did not lead to a significant reduction in the body weight of mice, similar to LH, but did result in a decrease in the weight of white adipose tissue and perinephric adipose tissue [22].

### 3.2. Effect of LH on Serum Lipid Levels in Mice

**Figure 3:**
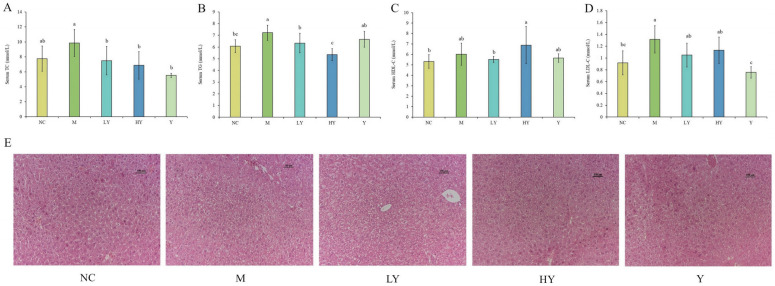
Effects of LH on blood lipid and liver injury in mice. (**A**) serum TC; (**B**) serum TG; (**C**) serum HDL-C; (**D**) serum LDL-C; (**E**) H&E staining of liver tissue. All data are shown as means ± SD (n = 10 per group). Different lowercase letters above the columns indicate statistically significant differences at *p* < 0.05 (one-way analysis of variance followed by Duncan post hoc test).

Elevated levels of TC, TG, and LDL-C, along with low or high levels of HDL-C can contribute to obesity and increase the risk of developing other chronic illnesses, and high levels of TC and TG are often seen in individuals with obesity [23,24]. The effects of different dosages of LH on blood lipids among groups are shown in Figure 3A–D. The mice in the M group exhibited abnormal levels of TC, TG, LDL-C, and HDL-C due to the high-fat diet. In comparison to the NC group, the M group showed an increase in all serum parameters, with HDL-C increasing in the HY group and TC, TG, and LDL-C decreasing in the LY and HY groups compared to the M group. Research has shown that increased HDL-C levels and decreased TC and TG levels can reduce the risk of cardiovascular disease [25]. Therefore, the findings suggest that LH could provide cardiovascular advantages. The study revealed that after 8 weeks of LH consumption, the levels of TC and TG in HFD mice were significantly reduced in a dose-dependent manner. While LH did not have a notable impact on serum LDL-C levels, there was still a decreasing trend that could be observed. In the meantime, there were no notable statistical variances observed between the NC and Y groups. These findings suggest that LH successfully regulated the blood lipid levels in mice fed a high-fat diet. 

### 3.3. Effects of LH on Liver Injury in Mice 

The liver plays an important role in lipid metabolism, and its damage could lead to lipid accumulation and a series of metabolic disorders [26]. The H&E-stained sections (Figure 3E) showed that the liver tissue structure of the NC group was intact, with hepatocytes organized in a neat cord-like pattern and no signs of steatosis. In the M group, liver cells showed small fat cavities and large fat droplets, indicating the presence of excessive hepatic fat, which is a characteristic feature of non-alcoholic fatty liver disease (NAFLD) [23]. Additionally, the size of the lipid droplets noticeably decreased following LH treatment, leading to a visible improvement in liver tissue damage compared to the M group. These results show that LH was effective in preventing liver injury and lipid accumulation and reducing the incidence of hepatic steatosis caused by HFD.

Non-alcoholic fatty liver disease (NAFLD) is the primary reason for chronic liver disease worldwide, often associated with various metabolic issues such as obesity, diabetes, dyslipidemia, and abnormal blood pressure [27,28]. Oxidative stress is positively associated with hepatocyte injury of NAFLD, while antioxidants could protect the cells or tissues against oxidative injury [29,30]. According to our previous results, LH was an efficient radical scavenger with relatively high antioxidant activity [18], which might explain why LH protected against HFD-induced liver damage, and it deserves further investigation.

### 3.4. Effect of LH on the Gut Microbiota Diversity and Composition in Mice

**Figure 4:**
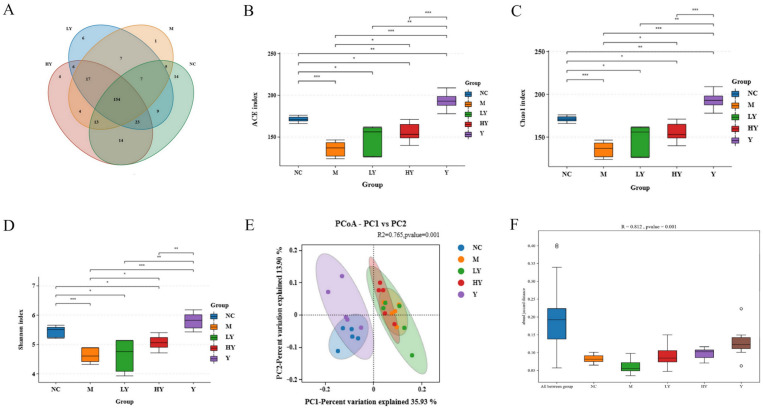
Effects of LH on the abundance and diversity of gut microbiota in mice. (**A**) Venn diagram; (**B**) ACE index; (**C**) Chao1 index; (**D**) Shannon index; (**E**) PCoA analysis; (**F**) Anosim analysis. n = 5 per group. * Data significant at *p* < 0.05, ** *p* < 0.01, *** *p* < 0.001 between groups (Student’s *t*-test).

Research has shown that the makeup of gut bacteria plays a role in the development of obesity [1]. Changes in diet, whether temporary or permanent, impact the composition of intestinal bacteria [31]. We conducted 16S rRNA sequencing on intestinal samples from the NC, M, LY, HY, and Y groups to investigate how changes in gut microbiota may be involved in LH’s supplementation of HFD-induced obesity. Following sequencing and filtering for quality, a total of 1,990,158 clean reads were obtained from the 25 samples (n = 5 per group), with each sample containing a minimum of 79,153 clean reads, leading to the discovery of 298 OTUs. Subsequently, a Venn diagram was applied to analyze the similarity and specificity of species distribution among groups (Figure 4A). There were 154 OTUs shared among the groups, with 14 unique OTUs in the NC group and 1 unique OTU in the M group. This suggests that the HFD decreases both the variety and distinctiveness of gut microbiota. Following LH treatment, the distinct OTU rose proportionally in mice fed a HFD, suggesting that LH therapy replenishes the diversity of species in the gut of overweight mice.

An increase in the gut microbiota abundance and diversity often predicts improvement of physical function [23]. Hence, Ace and Chao 1 indices (species richness) and the Shannon index (species diversity) were used to evaluate α-diversity among the samples. Figure 4B–D demonstrate that LH elevated the variety and complexity of the gut microbiota, leading to the mitigation of negative impacts from consuming a HFD. The diversity indices, including Chao 1, ACE, and Shannon, were significantly lower in the M group compared to the NC group (*p* < 0.001). However, after LH treatment, these indices gradually increased in a concentration-dependent manner (*p* < 0.05). Surprisingly, the Chao 1 index and ACE index were significantly increased in mice intestines when 5% LH was added to the normal diet (Y group), suggesting a notable rise in bacterial richness in normal mice caused by LH (*p* < 0.001).

The PCoA, based on β-diversity and utilizing the Jaccard distance metric, revealed a noticeable grouping of microbiota composition for each treatment group, which was found to be statistically significant according to ANOVA (Figure 4E, *p* < 0.01). Two distinct clusters were formed by the gut microbiota of the five sample groups. The distance between the NC and Y groups and the M, LY, and HY groups is relatively large. Furthermore, the ANOSIM test results (Figure 4F, *p* < 0.01) revealed a significant distinction between NC and M, which was notably diminished by LH treatment, indicating the beneficial effect of LH on the disrupted microbiota composition in obese mice.

**Figure 5:**
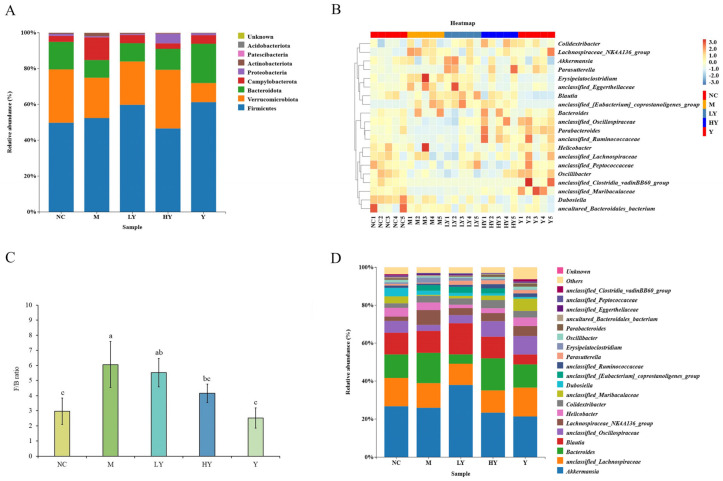
Effects of LH on the structure of gut microbiota in mice. (**A**) Structural changes in the gut microbiota at the phylum level; (**B**) heat map at the genus level; (**C**) ratio of Firmicutes to Bacteroidota (F/B); (**D**) structural changes in the gut microbiota at the genus level. Data of the F/B ratio are shown as means ± SD. Different lowercase letters above the columns indicate statistically significant differences at *p* < 0.05 (one-way analysis of variance followed by Duncan post hoc test). n = 5 per group.

Dysbiosis, an imbalance in the gut microbiome between helpful and harmful bacteria, can lead to intestinal oxidative stress, increased endotoxin levels, and compromised gut barrier function, ultimately leading to obesity [32]. Furthermore, numerous studies have clearly demonstrated a significant correlation between weight gain/obesity and the composition of gut microbiota [7,9]. A growing body of research has shown that mushroom supplements in diets effectively improve the gut microbiota composition [20,31]. Figure 5A displays the alterations in the gut microbiome among different groups at the phylum level. The gut microbiomes were primarily composed of Firmicutes, Verrucomicrobiota, Bacteroidota, Campylobacterota, and Proteobacteria, making up over 98% of the overall presence. The M group showed a notable reduction in the proportion of Bacteroidetes compared to the NC group (*p* < 0.05). To be specific, the abundance of Bacteroidetes was decreasing from 17.53% (NC) to 8.77% (M). After LH administration, the gut microbiota structure changed significantly. While there was no noticeable change in the prevalence of Firmicutes, Actinobacteriota decreased significantly (*p* < 0.05). Additionally, Bacteroidota and Proteobacteria increased, leading to a decrease in the Firmicutes-to-Bacteroidota ratio (F/B) after LH supplementation. This confirms that LH has an impact on the composition of the gut microbiota in mice fed a high-fat diet. Conversely, there were no significant differences in the relative abundances of gut microbiota at the gate level among the NC, HY, and Y groups. This suggests that using LH for dietary supplementation helped regulate gut microbiota abundances to be more similar to the NC group at this level, and high doses of LH helped reduce the dysbiosis of intestinal flora caused by HFD. Changes in the color gradient of the heat map (Figure 5B) could indicate an imbalance in the structure of the gut microbiota. The M group exhibited a lack of organization when compared to the NC group. After LH administration, the disordered flora structure was gradually close to the NC group. The findings indicate that LH successfully corrected the disruptions in the gut microbiome of overweight mice.

The elevated F/B ratio in the intestines of obese mice is a widely recognized factor closely linked to obesity induced by the HFD [33,34]. Consequently, the high-fat diet increased the Firmicutes-to-Bacteroidetes ratio significantly in the experimental group M, as shown in Figure 5C (*p* < 0.05). The F/B ratio in the LY group showed a slight decrease compared to the M group, with no significant difference, while it was significantly lower in the HY group. The decrease in level was in line with the rising LH addition. The values of F/B ratio among the NC, HY, and Y groups showed no significant differences. 

Figure 5D displays alterations in species composition within the top 20 genera based on their relative abundance. The study showed that the HFD treatment led to a notable reduction in the levels of unclassified *Oscillospiraceae*, unclassified *Muribaculaceae*, *Dubosiella*, unclassified *Ruminococcaceae*, *Parasutterella*, *Oscillibacter*, *Parabacteroides*, unclassified *Peptococcaceae*, and unclassified *Clostridia vadinBB60* group at the genus level (*p* < 0.05). Conversely, the levels of *Lachnospiraceae NK4A136* group, unclassified *Eubacterium coprostanoligenes* group, and *Erysipelatoclostridium* showed a significant increase following consumption of HFD (*p* < 0.05). Following LH supplementation in the HFD, there was a notable decrease in the *Lachnospiraceae NK4A136* group (*p* < 0.05), while unclassified *Muribaculaceae* showed a significant increase in the LY and HY groups (*p* < 0.05). There was a significant reduction in *Erysipelatoclostridium* levels in the HY group compared to the control group (*p* < 0.05). The Y group had the highest abundance of unclassified *Muribaculaceae*, *Oscillibacter*, and *Parabacteroides*, and the lowest level of *Dubosiella*, *Erysipelatoclostridium*, and *Blautia* (*p* < 0.05).

**Figure 6:**
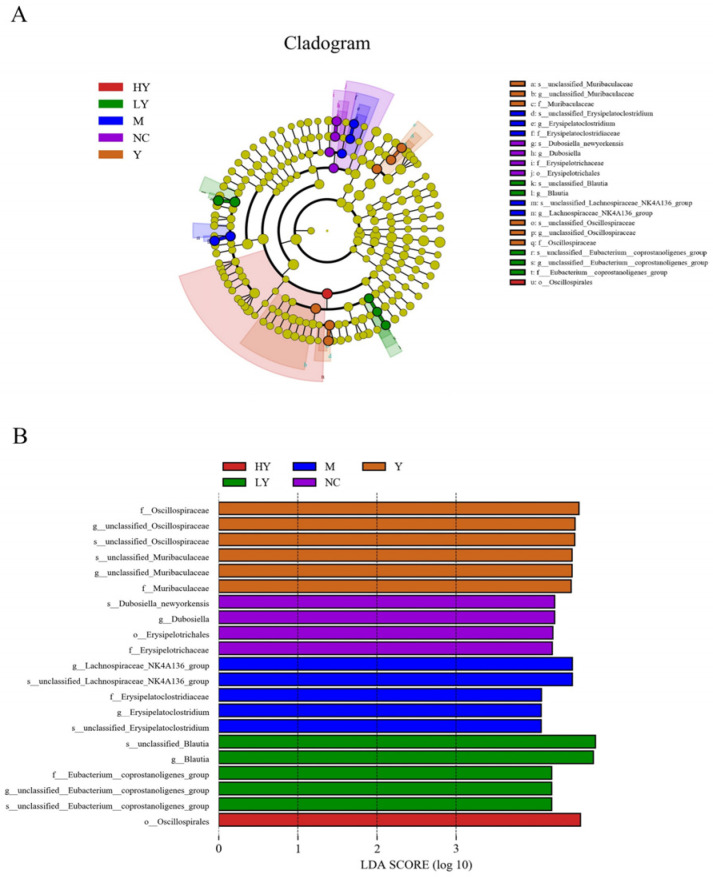
Effects of LH on gut microbiota at different developmental system levels in mice. (**A**) LEfSe analysis. (**B**) LDA score (>3.5). n = 5 per group.

To assess the impact of LH administration on the composition of the gut microbiome, a LEfSe comparison analysis was performed to identify biomarkers showing significant differences between groups. Figure 6A displayed taxa at various levels, showing significant distinctions (*p* < 0.05). Various species classifications are denoted by the circles. Circles, except for the yellow circles, indicate significant differences between species. The LDA score value showed significance when comparing microflora (Figure 6B). A total of 128 bacteria showed significant changes across the NC, M, LY, HY, and Y groups. The Y group exhibited the most phylotypes, with 19, while the M group had 6, the NC group had 5, the LY group had 5, and the HY group had 3. *Dubosiella* was the primary microbiota at the genus level in the NC group. The M group exhibited higher levels of *Lachnospiraceae NK4A136* group and *Erysipelatoclostridium* in comparison to the NC group (*p* < 0.05). *Blautia* and unclassified *Eubacterium coprostanoligenes* group in the LY group, along with unclassified *Ruminococcaceae* in the HY group, showed notable variances in relative prevalence (*p* < 0.05). Higher levels of *Oscillospiraceae*, unclassified *Muribaculaceae*, *Parabacteroides*, unclassified *Clostridia vadinBB60* group, *Faecalibaculum*, and *Oscillibacter* in the Y group were observed compared to the other groups.

**Figure 7:**
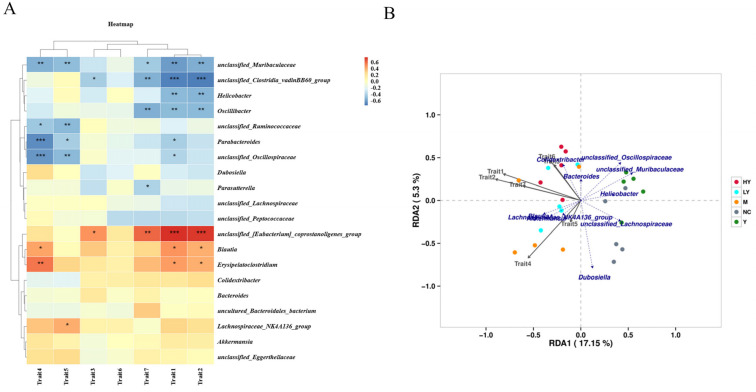
Correlation of obesity-related indices and gut microbiota at the genus level. (**A**) Correlation heat map of obesity-related indices and gut microbiota. * Data significant at *p* < 0.05, ** *p* < 0.01, *** *p* < 0.001. (**B**) RDA of obesity-related indices and gut microbiota at the genus level. Obesity-related indices are listed at the bottom of the panel: Trait 1, body weight; Trait 2, weight gain; Trait 3, liver weight; Trait 4, serum TC; Trait 5, serum TG; Trait 6, serum HDL-C; Trait 7, serum LDL-C.

Additionally, a Pearson correlation analysis was conducted to confirm the possible relationship between HFD consumption-induced alterations in gut microbiota composition and obesity-related indices (Figure 7). The weight of the mice showed a strong negative relationship with unclassified *Muribaculaceae*, unclassified *Clostridia vadinBB60* group, *Helicobacter*, *Oscillibacter*, *Parabacteroides*, and unclassified *Oscillospiraceae* (*r* = −0.619, *r* = −0.709, *r* = −0.523, *r* = −0.514, *r* = −0.429, and *r* = −0.398), while there was a significant positive correlation between the mice weight and unclassified *Eubacterium coprostanoligenes* group, *Blautia*, and *Erysipelatoclostridium* (*r* = 0.753, *r* = 0.481, and *r* = 0.453). The increase in weight was inversely related to unclassified *Muribaculaceae*, unclassified *Clostridia vadinBB60* group, *Helicobacter*, and *Oscillibacter* (*r* = −0.560, *r* = −0.720, *r* = −0.537, and *r* = −0.517), and positively associated with unclassified *Eubacterium coprostanoligenes* group, *Blautia*, and *Erysipelatoclostridium* (*r* = 0.732, *r* = 0.400, and *r* = 0.398). The unclassified *Clostridia vadinBB60* group (*r* = −0.433) and unclassified *Eubacterium coprostanoligenes* group (*r* = 0.484) revealed the opposite correlation with the liver weight. Serum TC and TG showed significant negative correlations with unclassified *Muribaculaceae*, unclassified *Ruminococcaceae*, *Parabacteroides*, and unclassified *Oscillospiraceae* (*r* = −0.548, *r* = −0.459, *r* = −0.674, and *r* = −0.638 for serum TC; *r* = −0.508, *r* = −0.522, *r* = −0.432, and *r* = −0.521 for serum TG), while TC had positive correlations with *Blautia* and *Erysipelatoclostridium* (*r* = 0.429 and 0.565). TG also had a positive correlation with *Lachnospiraceae NK4A136* group (*r* = 0.402). Additionally, a strong positive relationship was found between serum LDL-C levels and unclassified *Eubacterium coprostanoligenes group* (*r* = 0.604), while unclassified *Muribaculaceae*, unclassified *Clostridia vadinBB60* group, *Oscillibacter*, and *Parasutterella* showed negative correlations with this factor (*r* = −0.448, *r* = −0.550, *r* = −0.555, and *r* = −0.408). The results of the correlation analysis revealed the potential roles of these species in the pathogenesis of obesity. According to RDA, the most significant effect on gut microbiota was caused by weight gain compared to body weight, TC, LDL-C, HDL-C, liver weight, and TG, in that order (Figure 7B).

**Figure 8:**
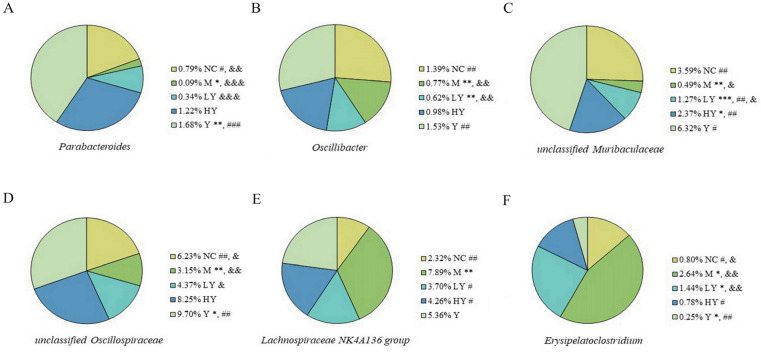
Relative abundance of specific genera of gut microbiota among groups. (**A**) Parabacteroides; (**B**) unclassified *Muribaculaceae*; (**C**) *Oscillibacter*; (**D**) unclassified *Oscillospiraceae*; (**E**) *Lachnospiraceae NK4A136* group; (**F**) *Erysipelatoclostridium*. * different from NC at *p* < 0.05; ** *p* < 0.01; *** *p* < 0.001. # different from M at *p* < 0.05; ## *p* < 0.01; ### *p* < 0.001. & different from HY at *p* < 0.05; && *p* < 0.01; &&& *p* < 0.001 (Student’s *t*-test). n = 5 per group.

Notably, we found that the abundances of *Parabacteroides*, unclassified *Muribaculaceae*, *Oscillibacter*, and unclassified *Oscillospiraceae* were decreased, whereas *Lachnospiraceae NK4A136* group and *Erysipelatoclostridium* were increased after HFD intake (for all, *p* < 0.05). Studies have shown that *Parabacteroides* is linked to lower rates of obesity [35,36], and introducing LH led to higher levels of *Parabacteroides* in both high-fat and normal diets (Figure 8A). Additionally, HFD reduced the abundance of *Oscillibacter*, but LH reshaped it (Figure 8B). Studies have indicated that *Oscillibacter* has a negative correlation with weight, epididymal fat index, and levels of TC and HDL-C [23]. In our study, this particular species showed a strong negative correlation with body weight, weight gain, and LDL-C levels. Meanwhile, unclassified *Muribaculaceae* and unclassified *Oscillospiraceae* also decreased in the M group (*p* < 0.01) but showed an increase following LH treatment in a manner that depended on the dosage (Figure 8C,D). The *Muribaculaceae* species, known for producing short-chain fatty acids (SCFAs), were found to have a protective effect against gut inflammation and play a key role in preventing or treating obesity [24,37]. Additionally, unclassified *Muribaculaceae* showed a negative association with body weight, weight gain, and levels of serum TC and TG in the current research. The unclassified *Oscillospiraceae* was discovered to be reduced in individuals induced by a high-fat diet and had a negative relationship with serum cholesterol levels [38], with a notable negative correlation between unclassified *Oscillospiraceae* and serum TC or TG. Conversely, the M group exhibited significantly elevated levels of *Lachnospiraceae NK4A136* group (*p* < 0.01) and *Erysipelatoclostridium* (*p* < 0.05). Reports indicate that the *Lachnospiraceae NK4A136* group could be a beneficial probiotic that plays a crucial part in maintaining gut balance and is inversely linked to obesity-related parameters [31,39]. Following LH treatment, there was a significant decrease in this species (*p* < 0.05), with no notable variation in the levels of *Lachnospiraceae NK4A136* group among the NC, LY, HY, and Y groups (Figure 8E). These results suggest that LH helps reduce the excessive presence of this species in the HFD-fed groups. *Erysipelatoclostridium* is known to be related to weight gain and various diseases, including obesity and colorectal cancer [40]. In this study, there was a strong positive correlation between *Erysipelatoclostridium* and body weight, weight gain, and serum TC. However, LH was able to reduce the levels of *Erysipelatoclostridium* in a dose-dependent manner. Thus, utilizing LH as a dietary supplement helped regulate the biological imbalance in mice fed a HFD and controlled the levels of the aforementioned microbiota towards a more typical state. 

## 4. Conclusions

In conclusion, our findings emphasize the beneficial impact of LH in preventing obesity caused by HFD and the ability of LH supplementation in diets to regulate gut microbiota in mice. However, further research is necessary to identify and determine the safety or applicable use of active compounds in LH that contribute to health benefits, explore the mechanism by which LH protects against HFD-induced liver damage, evaluate the functions of specific species in gut microbiota after LH intake, such as the production of SCFAs, and eventually provide a better understanding of the association between LH, gut microbiota, and host health. This study raises the possibility of LH use as a beneficial treatment for obesity, but more systematic work is warranted to elucidate the health benefits of LH in humans, which requires in vitro experiments in the human gut model or clinical trials before its applications as a routine therapy. Overall, the results provide a theoretical basis for the applications of LH and a good prospect for the development of LH as daily health food. 

## Data Availability

The original contributions presented in the study are included in the article, further inquiries can be directed to the corresponding author.
